# A novel mutation in the *STK11* gene causes heritable Peutz-Jeghers syndrome - a case report

**DOI:** 10.1186/s12881-017-0373-z

**Published:** 2017-02-23

**Authors:** Jing-Hui Chen, Jing-Jing Zheng, Qin Guo, Chao Liu, Bin Luo, Shuang-Bo Tang, Jian-Ding Cheng, Er-Wen Huang

**Affiliations:** 1 Department of Anesthesiology, Guangzhou Women and Children’s Medical Center, Guangzhou Medical University, Guangzhou, Guangdong China; 2 Faculty of Forensic Medicine, Zhongshan School of Medicine, Sun Yat-Sen University, No. 74 Zhongshan 2 Road, Guangzhou, 510080 China; 3 Department of Obstetrics and Gynecology, the First Affiliated Hospital of Guangdong Medical University, Guangzhou, Guangdong China; 4 Guangzhou Forensic Science Institute, Guangdong Provincial Key Laboratory of Forensic Genetics, Guangzhou, Guangdong China

**Keywords:** Frameshift mutation, Peutz-Jeghers syndrome, *STK11*, Truncating mutation, Case report

## Abstract

**Background:**

Peutz-Jeghers syndrome (PJS) is a rare disorder characterized by multiple gastrointestinal hamartomatous polyps and mucocutaneous pigmentation. *STK11* has been identified as a causative gene for this disease.

**Case presentation:**

Herein we report a Chinese Han kindred with PJS. Onset for the PJS signs in three of the patients was rarely as early as at birth. We identified a novel heterozygous mutation (c.440_441delGT, p.Arg147Leufs*15) in the gene *STK11*, causing a short frameshift followed by a deletion of 63% of the amino acids in the STK protein. This mutation co-segregated with the PJS phenotype, and was absent in two hundred of unrelated ethnicity-matched controls. The mutation led to expression decrease of unaffected STK11 protein in patients than in controls, as well in PJ polyps than in circulating leucocytes from the patients. Phosphorylation levels of the downstream kinase AMPKα altered according with the expression of STK11. These results indicated the possibility that haploinsufficiency and epigenetic reduction of STK11 contributed to the pathogenesis of the disease.

**Conclusion:**

This study identifies a novel mutation in the pathogenic gene *STK11* leading to PJS.

**Electronic supplementary material:**

The online version of this article (doi:10.1186/s12881-017-0373-z) contains supplementary material, which is available to authorized users.

## Background

Peutz-Jeghers syndrome (PJS) is a rare disorder characterized by multiple gastrointestinal hamartomatous polyps and mucocutaneous pigmentation [1, 2]. The disorder appears without gender predominance and is discovered in various ethnic groups, at an incidence estimated to range from 1/50,000 to 1/200,000 [3]. Clinical diagnosis of PJS can be made in a patient presenting one of the following signs: no less than two histologically confirmed PJ polyps, any number of PJ polyps with a family history of PJS, characteristic mucocutaneous pigmentation with a family PJS history, any number of PJ polyps associated with characteristic mucocutaneous pigmentation [4]. Mucocutaneous pigmented lesions usually occur at the lips, nostrils, perianal area, or digits; PJ polyps usually occur in the gastrointestinal tract, especially frequently in the small intestine, but have been also reported in extra-gastrointestinal organs [5, 6]. The polyps can lead to complications including alimentary canal obstruction, rectal prolapse, intussusception and severe bleeding with secondary anemia [7]. PJS increases the risk for various neoplasms, including gastrointestinal and extra-gastrointestinal, benign and malignant [8–10]. The serine/threonine kinase 11 coding gene (*STK11*) on chromosome 19p13.3 has been identified as a pathogenic gene of PJS [11, 12]. PJS signs usually appear within childhood or adolescent years [2, 3], and sometimes can also be firstly diagnosed in newborns or adults [13–15]. Here we report a Chinese Han family with PJS caused by a novel mutation (c.440_441delGT, p.Arg147Leufs*15) in the *STK11* gene.

### Subjects

**Fig. 1:**
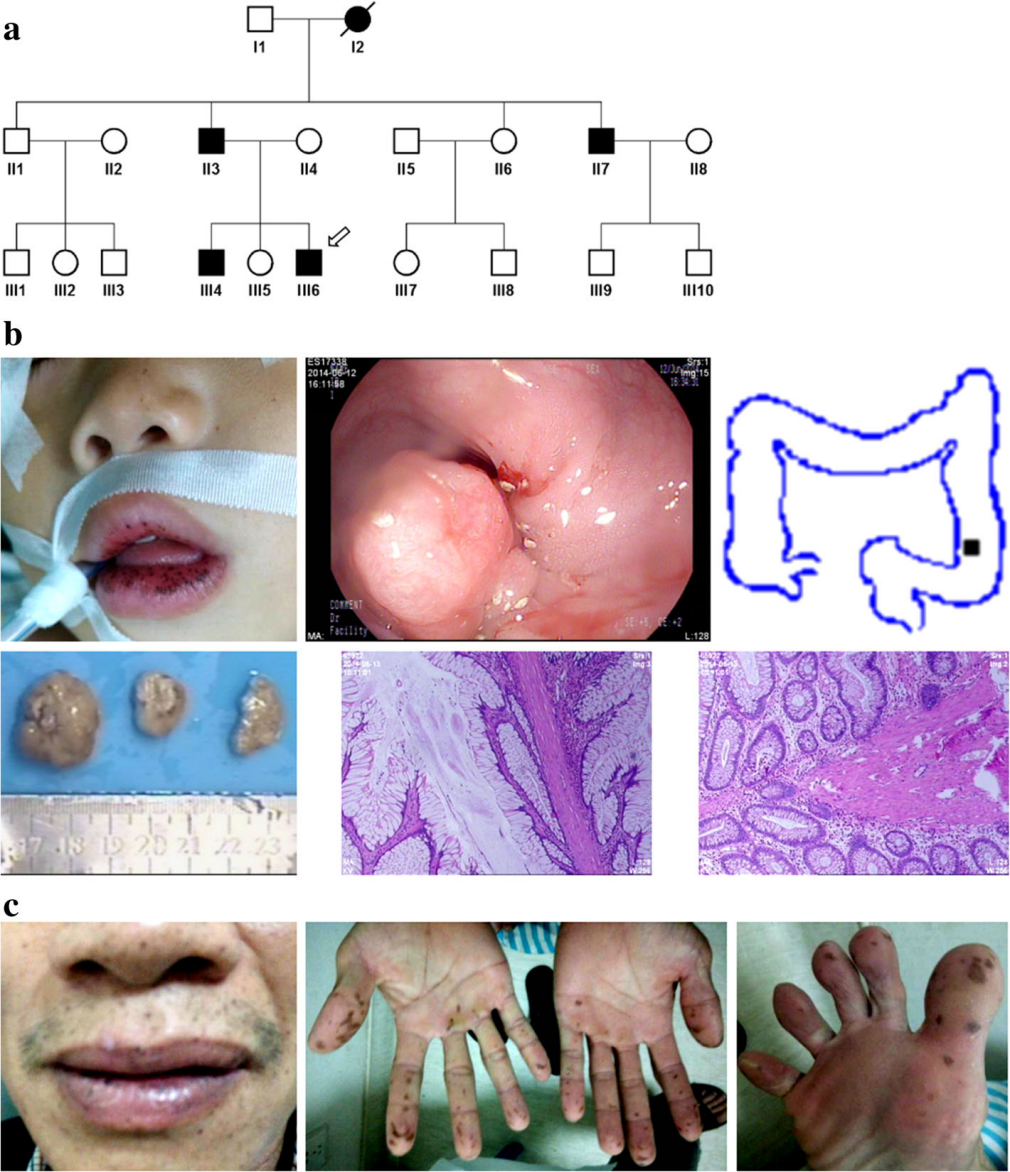
**a** Pedigree of the family with PJS. Square indicates male, circle indicates female. Filled and unfilled symbols indicate affected and unaffected individuals respectively. Slash indicates decedent. Arrow indicates the proband. **b** PJS signs the proband exhibited. Upleft, pigmentation at the lips; upper right, colonoscopy, filled square indicates location of the polyps in the descending colon; bottom left, polyps excised from the descending colon; bottom right, Representative hematoxylin-eosin-stained tissue slices of the largest polyp. **c** Sign of mucocutaneous pigmentation the proband’s father exhibited

The Chinese Han kindred with PJS was from Zhanjiang, Guangdong, China. Subjects in the kindred were diagnosed with or without PJS in Guangzhou Women and Children’s Medical Center. The proband III-6 received polyp surgery in the hospital mentioned above and the other patients did in hospitals in Zhanjiang (Fig. 1a). All of the unrelated Han controls were from Guangdong, not younger than 40 years, and diagnosed without PJS symptoms or family history in the First Affiliated Hospital of Guangdong Medical University.

## Case presentation

### Clinical presentation

Five patients distributed over three generations exhibited an autosomal dominant mode (Fig. 1a). The age of onset for mucocutaneous pigmentation was as early as at birth in III6, II7, III4, and at two years in II3, (Table 1, Fig. 1b and c). III6 was diagnosed with PJ polyps because of blood in stool and received intestinal surgery at the first year (Table 1). The PJ polyps relapsed and three hamartomatous polyps were excised from cavity of the descending colon at the age of 21 months (Fig. 1b). The largest polyp was 2.7 × 1.8 cm, looked smooth on the surface (Fig. 1b); its pedicle was 0.8 × 0.6 cm (data not shown). As shown in hematoxylin-eosin stained tissue slices, smooth muscle fibers from the muscularis mucosa extended into the inter-cryptas (Fig. 1b). Focal hemorrhage, necrosis and infiltration of a large number of inflammatory cells were observed (Fig. 1b). The proband’s father II3 exhibited pigmentation at both the lips and digits (Fig. 1c). His onset age was not defined but his father said it was before 2 years old. He was diagnosed with PJ polyps in the transverse colon at fifteen years (Table 1). III4, the elder brother of the proband, was diagnosed with PJ polyps in the rectum at two years (Table 1). The polyps extended out of the anus, as his parents described. II7, the uncle of the proband, was diagnosed with PJ polyps because of stomachache at sixteen years (Table 1). A polyp as large as the fist was surgically excised from the stomach, his families said. There is no determined information about the phenotypes of the grandmother I2 who died from neoplasm at forty. Her husband and oldest son told us that she suffered from pigmentation at the lips and digits, but the onset age was unknown. The grandfatherI1 is now eighty-three years old, and the PJS signs are still absent from him.

**Table 1:** Clinical features of the affected family members

ID	II3	II7	III4	III6	I2
Gender	male	male	male	male	female
Age (years)	46	38	13	4	Died of neoplasm at forty
Age at onset for mucocutaneous pigmentation (years)	earlier than 2	present at birth	present at birth	present at birth	unknown
Order of onset for mucocutaneous pigmentation	lips first, then digits	lips first, then digits	lips first, then digits	lips first, then digits	unknown
Age at first diagnosis of PJ polyps (years)	15	16	2	first year	unknown
Age at the first resection of polyps (years)	16	16	2	first year	unknown
Position of PJ polyps	transverse colon	stomach	rectum	descending colon	unknown

## Molecular studies

### Methods

#### Antibodies

Antibody (ab79355) targeting site 332–336 in STK11 was purchased from Abcam (Cambridge, UK). AMPKα-targeting antibaody (#2532) was purchased from Cell Signaling Technology (Boston, USA). GAPDH-targeting antibody (AF0006), phospho-AMPKα (Thr172)-targeting antibody (AA393), horseradish peroxidase-conjugated goat anti-rabbit secondary antibody (A0208), horseradish peroxidase-conjugated goat anti-mouse secondary antibody (A0216) were all purchased from Beyotime (Nantong, Jiangsu, China).

#### DNA preparation and sequencing of the *STK11* gene

Peripheral blood was collected in EDTA anticoagulant tubes. Genomic DNA was extracted from leukocytes using the Wizard Genomic DNA Purification Kit (Promega, Madison, WI, USA) according to the manufacturer’s instructions. 30 ng of DNA was used as PCR template to amplify the fragments of coding regions, 5′ and 3′ untranslated regions in *STK11*. Amplification products were Sanger sequenced using the ABI Genetic Analyzer (Applied Biosystems, Foster, CA, USA). The sequences of primers used for PCR and sequencing were listed in Additional file 1: Table S1.

#### Reverse transcription-polymerase chain reaction (RT-PCR) assay

Total RNA was extracted from leukocytes using the TRIzol reagent (Invitrogen, Carlsbad, CA, USA) according to the manufacturer’s protocol. RT-PCR assay was performed using the OneStep RT-PCR Kit (QIAGEN, Dusseldorf, Germany), according to the manufacturer’s protocol. RT-PCR primers were listed in Additional file 1: Table S2.

#### Protein extraction and western blot assay

Leukocytes isolated from circulating blood were lysed using RIPA lysis buffer (Beyotime, Nantong, Jiangsu, China) on ice. The lysis product was centrifuged at 12,000 × g for 10 min at 4 °C. The supernatant was collected for the following protein analysis. Tissue from the PJ polyps was cut into pieces and homogenized in RIPA lysis buffer (0.5 ml/100 mg tissue) for 10 min on ice. Then centrifuged as described above. Protein concentration was determined using the BCA reagent (Beyotime, Nantong, Jiangsu, China) according to the manufacturer’s instructions. The extracted proteins were isolated by SDS-PAGE electrophoresis in SDS-PAGE electrophoresis buffer (Beyotime, Nantong, Jiangsu, China) with 90 V voltage. The isolated proteins in SDS-PAGE gel were transfered onto polyvinylidene fluoride film in Western Transfer Buffer (Beyotime, Nantong, Jiangsu, China) with 200 mA current for 90 min. The polyvinylidene fluoride film with proteins was further treated as the following steps: incubated with 5% fat-free milk for 30 min and then with a primary antibody for 2 h at room temperature, washed to eliminate unspecific binding of the antibody, incubated with an horseradish peroxidase-conjugated secondary antibody for 1 h at room temperature, washed to eliminate unspecific binding of secondary antibody, incubated with BeyoECL chemiluminiscence reagent (Beyotime, Nantong, Jiangsu, China) according to the manufacturer’s instructions. Optical density representing the protein amount was captured and analyzed using the Luminescent Imaging Workstation (Tanon, Shanghai, China).

#### Hematoxylin-eosin staining of tissue slices

Paraffin-embedded polyp slices (5 μm of thick) were treated as the following steps: immersed in xylene for 3 × 2 min, dehydrated using alcohol at gradient concentrations (100%, 95%, 80%, 70%) for 5 × 2 min respectively, flushed with water for 3 min, incubated with Harris hematoxylin solution for 3 min, flushed with water for 3 min, incubated with 0.5% hydrochloric acid for 10 s, flushed with water for 15 min, incubated with alcohol (70%, 80% for 5 min respectively), incubated with 95% eosin-alcohol solution for 30 s, incubated with alcohol (95%, 100% for 5 min respectively), incubated with xylene-alcohol mixture (1:1) for 5 min, incubated with xylene for 5 min, sealed with neutral balsam and covered with coverslip.

### Results

**Fig. 2:**
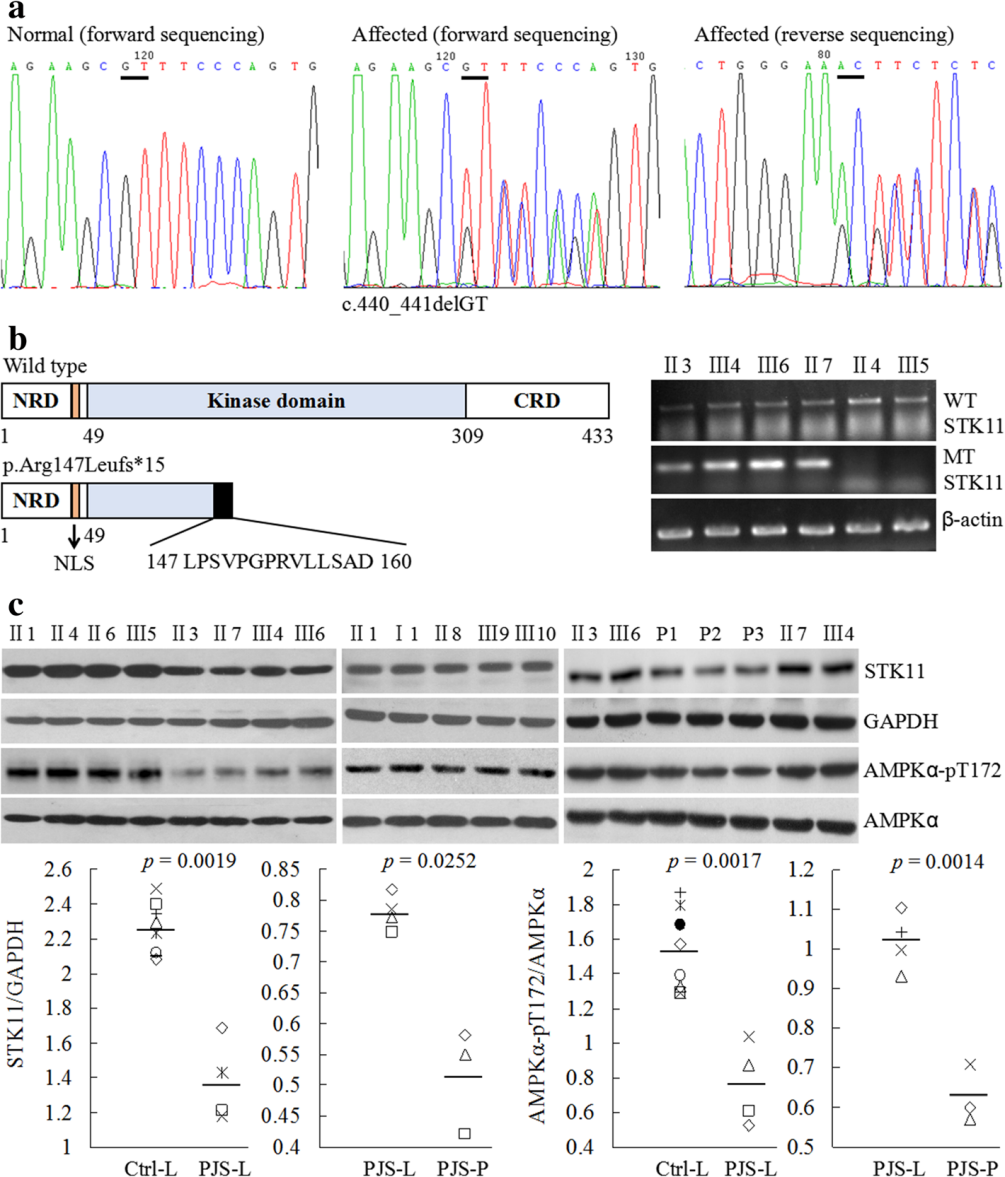
A novel mutation (c.440_441delGT, p.Arg147Leufs*15) in *STK11* was discovered. **a** Representative graphs of DNA sequencing surrounding the heterozygous mutation c.440_441delGT. **b** (Left) Schematics of the secondary structure or functional domains of the STK11 protein. NLS, Nuclear localization signal, NRD or CRD, N- or C-terminal regulatory domain. (Right) Images of the results of allele-specific reverse-transcription-polymerase chain reaction. WT, wild-type; MT, mutant. **c** Examination of the expression of unaffected STK11 protein, as well of the phosphorylation levels at threonine 172 in AMPKα. Ctrl-L, circulating leukocytes from controls (II1,II4, II6, III5,I1, II8, III9, III10); PJS-L, circulating leukocytes from the four patients (II3, II7, III4, III6); PJS-P, PJ polyps from III6. Unpaired 2-tailed Student’s t test was employed

We sequenced the exons, exon-intron boundaries, 5′ or 3′ untranslated region of the *STK11* gene in all of the family members. As a result, a heterozygous two-nucleotide deletion in exon three (c.440_441delGT) co-segregated with the PJS phenotypes (Fig. 2a). The mutation caused a frameshift at site 147 and a premature termination of translation at site 161 (p.Arg147Leufs*15, Fig. 2b). This mutation has not been reported in literatures or recorded in mutation databases. DNA from two hundred of unrelated ethnicity-matched normal individuals were sequenced and as a result the mutation was absent. Human STK11 protein comprises 433 residues, including a kinase catalytic domain (residues 49–309), N- and C-terminal regulatory domains, and a nuclear localization signal near the N-terminal (Fig. 2b). The frameshift led to a deletion of 57% of the catalytic domain and the whole C-terminal regulatory domain. We further studied the affection to mRNA using allele-specific reverse-transcription-polymerase chain reaction assay. As shown in Fig. 2b, all of the patients had a mutation-specific amplicon along with a wild-type-specific one, while two control family members just had the latter.

To study the functional affection of the mutation, we investigated STK11 protein expression in circulating leukocytes, using an antibody that targets residues 332–336 in STK11 and consequently reacts with the wild-type but not affected STK11. As a result, expression of the wild-type STK11 in controls was significantly higher than that in the patients (*p* = 0.0019, Fig. 2c), suggesting that the disease might result from STK11 haploinsufficiency. Interestingly, we found the wild-type STK11 expressed less in the three PJ polyps from patient III-6 than in circulating leukocytes from the four patients (*p* = 0.0252, Fig. 2c), suggesting that epigenetic reduction of STK11 expression might be a second contribution to polyps formation. AMPKα has been reported to be phosphorylated at threonine 172 by STK11 [16, 17]. Here we also observed that the phosphorylation levels of AMPKα paralleled with the levels of STK11 expression (Fig. 2c).

PJS onset in most reported patients was within childhood or adolescent years and it was reported that the median time to onset for gastrointestinal symptoms was 13 years of age [2, 15]. In this study, the age of onset for pigmentation in three of the patients was as early as at birth, not firstly but relatively rarely reported. The onset heterogeneity of PJS in this family was not confirmed. There is no determined information about the PJS onset in two of the patients. But it can’t exclude the possibility that the disease onset in the two patients was also as early as at birth. The heterogeneity in polyps location in spite of the same mutation remains to be studied.

As a positive regulation, STK11 is released and shuttles from nucleus to cytoplasm, and interacts with STRADα/β and MO25α/β to stabilize the kinase activity [16, 18]. The entire kinase catalytic domain and amino acids 319–343 in STK11 are necessary for binding to STRAD [18]. So it is self-evident that the mutant identified here is unable to associate with the STRADα/β-MO25α/β complex. Other interacting partners of STK11 have been successively identified and the indispensable motifs in STK11 for interacting with some of them have not yet been determined [19]. So we can’t exclude the possibility that dominant negative effect is a third molecular mechanism by which the mutation results in PJS, if the inactive mutant occupies the partners.

## Discussion

There are hundreds of mutations in the STK11 gene reported to cause PJS. Here we identified a novel mutation which leads to a truncated STK11 protein, functionly causes inherited, even early-onset (remained to be further confirmed) PJS. Epigenetic expression reduction might be also one of the pathogenic mechanisms caused by the mutation. This study further expanded the mutation spectrum of PJS.

## Conclusion

In conclusion, in this study we identified a novel heterozygous mutation (c.440_441delGT, p.Arg147Leufs*15) in the *STK11* gene causing the inherited PJS in this family. Haploinsufficiency and epigenetic expression reduction of STK11 might contribute to the pathogenesis of the disease.

## Additional file

Additional file 1: Table S1. Sequences of the primers for PCR and sequencing of the *STK11* gene. **Table S2.**. Sequences of the RT-PCR primers. (DOC 31 kb)

## Abbreviations

AMPKα: AMP-activated catalytic subunit alpha
MO25α/β: Mouse protein 25 alpha/beta
PJS: Peutz-Jeghers syndrome
STK11: Serine/threonine kinase 11
STRADα/β: STE20-related kinase adaptor alpha/beta

## References

[1] Jeghers H, Mc KV, Katz KH (1949). Generalized intestinal polyposis and melanin spots of the oral mucosa, lips and digits; a syndrome of diagnostic significance. N Engl J Med.

[2] Westerman AM, Entius MM, de Baar E (1999). Peutz-Jeghers syndrome: 78-year follow-up of the original family. Lancet.

[3] Giardiello FM, Trimbath JD (2006). Peutz-Jeghers syndrome and management recommendations. Clin Gastroenterol Hepatol.

[4] Aaltonen LA (2000). Hereditary intestinal cancer. Semin Cancer Biol.

[5] Sommerhaug RG, Mason T (1970). Peutz-Jeghers syndrome and ureteral polyposis. JAMA.

[6] Vogel T, Schumacher V, Saleh A, Trojan J, Moslein G (2000). Extraintestinal polyps in Peutz-Jeghers syndrome: presentation of four cases and review of the literature. Deutsche Peutz-Jeghers-Studiengruppe. Int J Colorectal Dis.

[7] Beggs AD, Latchford AR, Vasen HFA (2010). Peutz-Jeghers syndrome: a systematic review and recommendations for management. Gut.

[8] Boardman LA, Thibodeau SN, Schaid DJ (1998). Increased risk for cancer in patients with the Peutz-Jeghers syndrome. Ann Intern Med.

[9] Young RH (2005). Sex cord-stromal tumors of the ovary and testis: their similarities and differences with consideration of selected problems. Mod Pathol.

[10] van Lier MGF, Westerman AM, Wagner A (2011). High cancer risk and increased mortality in patients with Peutz-Jeghers syndrome. Gut.

[11] Jenne DE, Reimann H, Nezu J (1998). Peutz-Jeghers syndrome is caused by mutations in a novel serine threonine kinase. Nat Genet.

[12] Hemminki A, Markie D, Tomlinson I (1998). A serine/threonine kinase gene defective in Peutz-Jeghers syndrome. Nature.

[13] Fernandez Seara MJ, Martinez Soto MI, Fernandez Lorenzo JR, Trabazo S, Gamborino E, Forteza VJ (1995). Peutz-Jeghers syndrome in a neonate. J Pediatr.

[14] Al Faour A, Vrsansky P, Abouassi F, Dabbagh H, Gross P, Retbi JM (2002). Peutz-Jeghers colonic tumour in a newborn. Eur J Pediatr Surg.

[15] Amos CI, Keitheri-Cheteri MB, Sabripour M (2004). Genotype-phenotype correlations in Peutz-Jeghers syndrome. J Med Genet.

[16] Hawley SA, Boudeau J, Reid JL (2003). Complexes between the LKB1 tumor suppressor, STRAD alpha/beta and MO25 alpha/beta are upstream kinases in the AMP-activated protein kinase cascade. J Biol.

[17] Shaw RJ, Kosmatka M, Bardeesy N (2004). The tumor suppressor LKB1 kinase directly activates AMP-activated kinase and regulates apoptosis in response to energy stress. Proc Natl Acad Sci U S A.

[18] Baas AF, Boudeau J, Sapkota GP (2003). Activation of the tumour suppressor kinase LKB1 by the STE20-like pseudokinase STRAD. EMBO J.

[19] Zhan YY, Chen Y, Zhang Q (2012). The orphan nuclear receptor Nur77 regulates LKB1 localization and activates AMPK. Nat Chem Biol.

