# Supplementary material for: A novel mutation in the STK11 gene causes heritable Peutz-Jeghers syndrome - a case report
Source: BMC Med Genet. 2017 Feb 23;18:19. doi: 10.1186/s12881-017-0373-z (PMC5324205; doi:10.1186/s12881-017-0373-z)
Supplement: Additional file 1: Table S1. — Sequences of the primers for PCR and sequencing of the STK11 gene. Table S2.. Sequences of the RT-PCR primers. (DOC 31 kb) [file 12881_2017_373_MOESM1_ESM.doc]

**A novel mutation in the *STK11* gene causes heritable Peutz-Jeghers syndrome - a case report**

Jing-Hui Chen^1*^, Jing-Jing Zheng^2*^, Qin Guo^3*^, Chao Liu^2,4*^, Bin Luo^2^, Shuang-Bo Tang^2^, Jian-Ding Cheng^2^, Er-Wen Huang^2^

^1^Department of Anesthesiology, Guangzhou Women and Children's Medical Center, Guangzhou Medical University, Guangzhou, Guangdong, China

^2^Faculty of Forensic Medicine, Zhongshan School of Medicine, Sun Yat-Sen University,Guangzhou, Guangdong, China

^3^Department of Obstetrics and Gynecology, the First Affiliated Hospital of Guangdong Medical University, Guangzhou, Guangdong, China

^4^Guangzhou Forensic Science Institute, Guangdong Provincial Key Laboratory of Forensic Genetics, Guangzhou, Guangdong, China

^*^ These authors contributed equally to this work

Correspondence to Er-Wen Huang, No. 74 Zhongshan 2 Road, Guangzhou 510080, China, e-mail huangerw@mail.sysu.edu.cn; or to Jian-Ding Cheng, No. 74 Zhongshan 2 Road, Guangzhou 510080, China, e-mail chengjd@mail.sysu.edu.cn.

Table S1 Sequences of the primers for PCR and sequencing of the *STK11* gene

| Fragment | Forward primer | Reverse primer |
| --- | --- | --- |
| 5’UTR+Exon1 | 5'-GGACCGGACGCTGAGGGCACT-3' | 5'-CGGGTCCAGCTCAGGGTGTTAAGA-3' |
| Exon2 | 5'-TGTCCTAACTGTGTCCTCCA-3' | 5'-ATTGCCACAATGGCTGACTT-3' |
| Exon3 | 5'-GGGCGTGTCCTCGTGTCATCTGTG-3' | 5'-GCCGGCTGCACCCCAAAGTCT-3' |
| Exon4+5 | 5'-CCTAGCCTTTCCTCTGTCCT-3' | 5'-CACCATCTGCCGTATGAGTT-3' |
| Exon6 | 5'-GTGGAAGGTGGTGAAGACAG-3' | 5'-CACTCAGTCCTCTCAATGCC-3' |
| Exon7 | 5'-CCTTAGGAGCGTCCAGGTA-3' | 5'-CGCATGTCCTCACTCAGAC-3' |
| Exon8 | 5'-GACATGGCTGAGCTTCTGTG-3' | 5'-CTTTGGGGACGTGGGATTG-3' |
| Exon9 | 5'-GCAGCATTTCAGGCTGGATA-3' | 5'-GGTCACCATGACTGACTAGC-3' |
| 3’UTR | 5'-AGCCCATCCACCAGCGTCAGAG-3' | 5' CCGACCTCCGAGACCGTTTACC 3' |

Table S2 Sequences of the RT-PCR primers

| Gene | Forward primer | Reverse primer |
| --- | --- | --- |
| Normal *STK11* | 5'-GGCCGTCAAGATCCTCAAGAAG-3' | 5'-GGGCCTGGCACACTGGGAAA-3' |
| Affected *STK11* | 5'-GGCCGTCAAGATCCTCAAGAAG-3' | 5'-GGGCCTGGCACACTGGGAAG-3' |
| *ACTB* | 5'-GGACTTCGAGCAGGAGATGG-3' | 5'-GCACCGTGTTGGCGTAGAGG-3' |
